# Youth vaping and smoking and parental vaping: a panel survey

**DOI:** 10.1186/s12889-020-09228-w

**Published:** 2020-07-28

**Authors:** Michael J. Green, Linsay Gray, Helen Sweeting

**Affiliations:** MRC/CSO Social and Public Health Sciences Unit, University of Glasgow, 200 Renfield Street, Glasgow, G2 3AX UK

**Keywords:** E-cigarettes, Smoking, Youth, Parents

## Abstract

**Background:**

Concerns remain about potential negative impacts of e-cigarettes including possibilities that: youth e-cigarette use (vaping) increases risk of youth smoking; and vaping by parents may have impacts on their children’s vaping and smoking behaviour.

**Methods:**

With panel data from 3291 youth aged 10–15 years from the 7th wave of the UK Understanding Society Survey (2015–2017), we estimated effects of youth vaping on youth smoking (ever, current and past year initiation), and of parental vaping on youth smoking and vaping, and examined whether the latter differed by parental smoking status. Propensity weighting was used to adjust for measured confounders and estimate average effects of vaping for all youth, and among youth who vaped. E-values were calculated to assess the strength of unmeasured confounding influences needed to negate our estimates.

**Results:**

Associations between youth vaping and youth smoking were attenuated considerably by adjustment for measured confounders. Estimated average effects of youth vaping on youth smoking were stronger for all youth (e.g. OR for smoking initiation: 32.5; 95% CI: 9.8–107.1) than among youth who vaped (OR: 4.4; 0.6–30.9). Relatively strong unmeasured confounding would be needed to explain these effects. Associations between parental vaping and youth vaping were explained by measured confounders. Estimates indicated effects of parental vaping on youth smoking, especially for youth with ex-smoking parents (e.g. OR for smoking initiation: 11.3; 2.7–46.4) rather than youth with currently smoking parents (OR: 1.0; 0.2–6.4), but these could be explained by relatively weak unmeasured confounding.

**Conclusions:**

While measured confounding accounted for much of the associations between youth vaping and youth smoking, indicating support for underlying propensities, our estimates suggested residual effects that could only be explained away by considerable unmeasured confounding or by smoking leading to vaping. Estimated effects of youth vaping on youth smoking were stronger among the general youth population than among the small group of youth who actually vaped. Associations of parental vaping with youth smoking and vaping were either explained by measured confounding or could be relatively easily explained by unmeasured confounding.

## Background

Use of electronic cigarettes (e-cigarettes) has been rising since 2011 in the United Kingdom (UK) [1] and internationally [2], though has recently plateaued in the UK [3, 4]. We refer here to use of e-cigarettes as ‘vaping’, considered distinct from ‘smoking’ traditional cigarettes. Internationally, concerns have been raised, particularly relating to youth, that because the behaviours are similar and many (but not all) e-cigarettes contain nicotine, vaping behaviour could help establish and/or maintain smoking behaviour [5–9]. As vaping prevalence has risen among adults, it is also important to understand the impacts this may have on young people who live with those adults, for example, the impacts on the children if a smoking parent were to switch to vaping. While vaping may be safer than smoking, nicotine exposure in adolescence specifically, may still have some concerning consequences, including: increased risk for developing psychiatric disorders, effects on brain development and later-life cognition, and priming for future substance abuse [10].

The notion that vaping increases risk for smoking can be contrasted with that of common liabilities [8, 9]: that underlying propensities for both behaviours account for their close association among youth [2, 6, 11–16]. While many studies [2, 11–14] have adjusted for measured differences in background factors, unmeasured common liabilities remain possible explanations [8, 17], even for longitudinal studies showing vaping preceding smoking [6, 15, 18–22] and where respondents had stated no intention of smoking [23]. Common liabilities and vaping increasing smoking risk (or indeed, smoking increasing vaping risk [22]) are not mutually exclusive explanations for associations between vaping and smoking among youth. It is more important to establish the relative contribution of each in explaining associations between youth smoking and vaping, than to try and establish any one as the ‘true’ explanation.

Parental smoking is among the most established risk factors for youth smoking [24], so it seems likely parental vaping could also influence youth behaviour, though common liabilities are also viable explanations for associations between parental vaping and youth behaviour. A UK study has shown associations between parental vaping and youth initiation of smoking and vaping [22], though these were not the main focus of their study and were attenuated in adjusted models. If there are effects of parental vaping, these could differ depending on parental smoking status. If e-cigarettes are viewed as an aid to smoking cessation [3, 25] then parental use could make smoking seem less normative and reduce risk of smoking initiation, especially if parents completely switch from smoking to vaping. On the other hand, dual use (of cigarettes and e-cigarettes) by parents could result in the behaviours appearing to youth as linked and complementary and increase risk for both. Indeed, a study in Mexico showed youth susceptibility to vaping and smoking to be higher where family members were either cigarette or dual users, but not where family members only vaped [26].

Understanding of these issues may be helped by more clearly defining the effects of interest [27], recognising that vaping is not random and there may be important differences between those who do and do not vape. Imagine two hypothetical (and unethical) trials in which youth were randomised to either vape or not vape. In the first, interest is in randomising among all youth, with any resulting difference in smoking interpreted as an average treatment effect (ATE), i.e. the average effect of vaping among all youth. In the second, randomisation is performed among youth who do vape (who may have considerably different characteristics from the general population of youth), with any difference in smoking representing the average treatment effect among the treated (ATT), i.e. the average effect of not preventing those who do vape from doing so. Similarly, for parental vaping, the ATE would be the average effect of parental vaping among all youth, while ATTs represent effects among the group of youth whose parents actually vaped. These estimates will be identical if effects are uniform, but may differ if effects are heterogeneous, i.e. varying with the background factors that predict vaping. Both estimates offer useful insights: ATEs indicate potential impacts if vaping were adopted more widely, while ATTs can indicate what may happen if interventions were implemented to reduce existing vaping behaviour.

With data from a large survey designed to be representative of UK households, we use propensity weighting [27] to estimate ATE and ATT effects of youth vaping on youth smoking and of parental vaping on youth smoking and vaping. For parental vaping, we conduct analyses for all youth, but also stratify by parental smoking status.

## Methods

### Sample

Respondents were from the 7th Wave of Understanding Society, a panel survey based on annual interviews conducted within UK households [28]. In fieldwork spanning 2015–2017, 4534 youth aged 10–15 were eligible for inclusion because they lived in a household with a member of the (adult) study sample. Youth were not interviewed directly but confidentially filled in a self-completion questionnaire after their parent or carer had given permission for them to take part. A total of 3635 youth (80.2% of those eligible) in 2759 households completed questionnaires. Valid survey weights designed to account for household attrition, non-response and over-sampling were available for 3291 youth (90.5% of those responding) and used throughout to render the sample representative of the UK [29]. Multiple imputation of missing values (25 datasets, using an unconstrained model in which all analysis variables predicted all others) enabled inclusion of all observed data from respondents with valid weights [30] (proportions missing for most variables were between 0 and 5.3%, though 18.6% had missing data for ethnicity; see Table 1).

**Table 1:** Sociodemographic patterning of youth vaping and smoking

	Observed N (%)	Missing N (%)	Imputed N (%)	Current Vaping		Ever Smoker		Current Smoker		Smoking Initiation (*N* = 3075^a^)	
				Yes %	*P*-Value	Yes %	*P*-Value	Yes %	*P*-Value	Yes %	*P*-Value
All	3291 (100.0)	0 (0.0)	3291 (100.0)	3.4	–	7.4	–	2.3	–	0.9	–
No Vaping	3069 (96.5)	112 (3.4)	3179 (96.6)			5.5	< 0.001	1.2	< 0.001	0.5	< 0.001
Current Vaping	110 (3.5)		112 (3.4)			63.3		31.9		24.1	
Never Smoker	3022 (92.6)	27 (0.8)	3047 (92.6)	1.4	< 0.001						
Ever Smoker	242 (7.4)		244 (7.4)	29.0							
Non Smoker	3190 (97.7)	27 (0.8)	3216 (97.7)	2.4	< 0.001						
Current Smoker	74 (2.3)		75 (2.3)	47.5							
Never Smoker^a^	3022 (99.1)	27 (0.8)	3046 (99.1)	1.4	< 0.001						
Initiating Smoker^a^	28 (0.9)		29 (0.9)	45.8							
Male	1629 (49.5)	0 (0.0)	1629 (49.5)	4.2	0.019	7.5	0.859	2.4	0.626	1.2	0.207
Female	1662 (50.5)		1662 (50.5)	2.7		7.3		2.2		0.7	
Age 10	520 (15.8)	0 (0.0)	520 (15.8)	0.8	< 0.001	0.0	< 0.001	0.0	< 0.001	0.0	< 0.001
Age 11	596 (18.1)		596 (18.1)	0.0		1.8		0.2		0.2	
Age 12	561 (17.0)		561 (17.0)	0.7		1.8		0.2		0.2	
Age 13	493 (15.0)		493 (15.0)	2.7		5.7		0.8		0.7	
Age 14	588 (17.9)		588 (17.9)	6.9		14.6		4.7		3.1	
Age 15	533 (16.2)		533 (16.2)	9.2		20.6		7.7		1.7	
England	2830 (86.0)	1 (0.0)	2831 (86.0)	3.4	0.618	7.4	0.892	2.1	0.311	0.8	0.080
Wales	111 (3.4)		111 (3.4)	2.7		6.4		1.8		1.0	
Scotland	263 (8.0)		263 (8.0)	3.0		7.6		3.8		2.4	
Northern Ireland	86 (2.6)		86 (2.6)	5.8		9.3		3.5		0.2	
White UK	2269 (84.7)	612 (18.6)	2764 (84.0)	3.6	0.196	7.9	0.018	2.4	0.168	1.0	0.217
Ethnic Minority	410 (15.3)		527 (16.0)	2.4		4.7		1.4		0.4	
Couple Parents	2437 (75.1)	45 (1.4)	2472 (75.1)	2.6	< 0.001	5.8	< 0.001	1.6	< 0.001	0.7	0.011
Single Parent	809 (24.9)		819 (24.9)	5.9		12.2		4.3		1.8	
Parents Never Smokers	1154 (35.6)	53 (1.6)	1174 (35.7)	3.0	0.118	5.7	0.010	1.3	0.002	0.9	0.896
Ex-Smoking Parent(s)	1282 (39.6)		1300 (39.5)	3.0		7.9		2.2		1.0	
Current Smoking Parent(s)	802 (24.8)		817 (24.8)	4.6		9.2		3.8		1.0	
No Parental Vaping	2857 (88.2)	53 (1.6)	2903 (88.2)	3.1	0.014	6.8	< 0.001	2.0	0.019	0.8	0.004
Parental Vaping	381 (11.8)		388 (11.8)	5.6		12.3		4.0		2.4	
Parental Education -Degree	1697 (53.0)	87 (2.7)	1741 (52.9)	3.0	0.386	6.3	0.017	1.4	< 0.001	0.6	0.137
A-Level or equivalent	658 (20.5)		674 (20.5)	3.5		7.3		2.3		1.3	
GSCE or equivalent	759 (23.7)		783 (23.8)	4.3		9.3		3.9		1.4	
No Qualifications	90 (2.8)		93 (2.8)	3.3		12.7		4.5		0.2	
Managerial/Professional	1282 (41.1)	173 (5.3)	1345 (40.9)	1.8	< 0.001	6.2	0.118	1.4	0.006	0.5	0.117
Intermediate	461 (14.8)		490 (14.9)	4.4		7.3		2.5		1.0	
Routine	510 (16.4)		536 (16.3)	5.7		8.2		2.0		1.7	
Not employed	865 (27.7)		920 (28.0)	3.8		8.8		3.7		1.1	
Highest Income Quartile	463 (14.3)	48 (1.5)	472 (14.3)	1.9	< 0.001	5.9	< 0.001	1.4	< 0.001	0.9	0.005
2nd Quartile	836 (25.8)		851 (25.9)	2.1		6.8		2.0		0.3	
3rd Quartile	1079 (33.3)		1094 (33.2)	3.5		6.0		1.4		0.7	
Lowest Income Quartile	865 (26.7)		874 (26.6)	5.5		10.7		4.2		1.9	

^a^Adolescents who had reported smoking in previous waves of the survey were excluded here (*n* = 216), so percentages indicate the proportion of those who had never smoked before who were current smokers in this wave of the survey

### Measures

Youth and parents self-reported vaping in response to the question: “Do you ever use electronic cigarettes (e-cigarettes)?” (Yes/No). This was the first survey wave in which respondents had been asked about vaping. However, smoking was self-reported by youth in this and up to five earlier waves of the survey, depending on when they had reached age 10. Youth were first asked “Do you ever smoke cigarettes at all?” (yes/no), and if ‘yes’ were asked to tick a statement that best described them (only smoked once or twice; used to smoke but don’t now; sometimes smoke but not every week; usually smoke between one and six cigarettes a week; usually smoke more than 6 cigarettes a week). Youth responding ‘no’ in wave 7 and all previous waves for which data were available were coded as ‘never smoked’, while a ‘yes’ response in any wave was coded as having ‘ever smoked’. Current smoking was coded as smoking sometimes or more frequently at wave 7. Initiation of smoking was coded as current smoking with no indication of smoking in earlier survey waves. Parents were asked about current smoking (yes/no) in wave 7 and questions on smoking history from previous survey waves were used to distinguish never from ex-smokers. Parental smoking (never, ex, current) and vaping (yes/no) were coded according to the highest level of use from either parent, i.e. parental current smoking/vaping indicates that at least one parent smoked or vaped, while parental ex-smoking indicates no parents smoked currently but at least one was an ex-smoker.

Socioeconomic position (SEP) was measured with three variables at the household level (taking the more advantaged responses from couple parents): highest educational level (degree or higher, A-Level or equivalent, GCSE or equivalent, or no qualifications); occupational status using NS-SEC codes (managerial or professional, intermediate, routine, or not employed); and household income, equivalised for household composition and split into quartiles. For ease of presentation, SEP measures were treated as ordinal when assessing confounder balance, with higher values indicating greater socioeconomic disadvantage. Indicators of gender (male vs female), ethnicity (White UK vs ethnic minority), family structure (couple vs single parents), UK country (England, Wales, Scotland, and Northern Ireland) and interview date (to account for temporal trends in smoking and vaping during fieldwork) were also included, with youth age (in years) as a continuous variable.

### Statistical analyses

We estimated ATEs and ATTs using a propensity weighting procedure, which is designed to balance measured confounders across the main exposure groups, i.e. youth who did and did not vape, and youth with parents who did and did not vape [27, 31]. This involves first running logistic regression models to predict each exposure, based on measured confounders (identified a priori). Gender, age, ethnicity, family structure, household SEP, UK country and interview date were treated as potential confounders throughout, as was parental smoking (except when stratifying on this variable). For estimating effects of youth vaping, parental vaping was included as an additional confounder.

The predicted probability of each individual’s observed exposure status was used to calculate weights for estimating ATEs and ATTs. Table 2 details these calculations. ATE weights re-weight exposed and unexposed respondents to resemble the total sample (with regards to measured confounders), while ATT weights re-weight the unexposed respondents to resemble the exposed group. Prior to using these weights to estimate effects, validity of the weights was assessed by examining mean differences in confounders associated with the relevant exposure [31]. Weights were deemed valid if confounder differences, expressed in standard deviation units, were reduced close to zero (with differences < 0.2 considered close to 0). Models predicting exposure probability initially used main effects of confounders only, but where imbalance remained, the model was revised by adding interactions terms and then re-assessed. Improvements in confounder balance from model revisions were balanced against sufficient overlap of propensity distributions between exposed and unexposed groups by confirming that mean ATE weights were close to 1 [31, 32] (the same is not expected of mean ATT weights). Deviations from this would suggest that some individuals were being assigned extreme weights, indicating risk of making inferences not strongly supported by the available data.

**Table 2:** Calculation and interpretation of propensity weights

Estimand	How predicted probabilities of exposure are used to calculate weights^a^:		Re-weighting of confounding characteristics in exposure groups:		Estimated effect for youth vaping applies to …	Estimated effect for Parental Vaping applies to …
	Numerator	Denominator	Unexposed	Exposed		
Average Treatment Effect (ATE)	P	P^	Resemble sample characteristics	Resemble sample characteristics	All youth	All youth
Average Treatment Effect among the Treated (ATT)	1 if exposed, 1-P^ if unexposed	1 if exposed, P^ if unexposed	Resemble Exposed group	Unchanged	Youth who do vape	Youth whose parents did vape

P=Overall, unadjusted probability of individual’s observed exposure level

P^=Predicted probability of individual’s observed exposure level conditional on confounders

^a^When investigating effects of parental vaping within strata of parental smoking, both P and P^ were additionally conditional on parental smoking

ATEs and ATTs were then estimated in weighted logistic regressions of each outcome on the exposure of interest. For comparison, we also present associations weighted for sample selection only (labelled “sample weighted associations”). Standard errors were adjusted for clustering of youth within households. Z-tests were used to compare differences in effect estimates between strata of parental smoking and between ATEs and ATTs [33].

Analyses of smoking initiation excluded 216 youth who had reported ever smoking in previous survey waves. These prior smokers were older, more likely to be vaping and to have single parents. Since this could introduce selection bias, these differences were reduced by additional weighting back to the total sample for analyses of initiation.

Since these estimates may still be biased by unmeasured confounding, we calculate e-values for each point estimate and for the lower limit of the confidence interval [34]. E-values represent the minimum strength of association (OR in our analysis) that a set of unmeasured confounders would need to have with both the outcome and exposure of interest (independent of measured confounders), in order to respectively explain away the association, or cause its lower confidence interval to include the null (if it already includes the null the e-value for the lower limit will be 1). We include e-values for the sample-weighted associations, to indicate how much these were reduced by weighting for measured confounders.

## Results

Sociodemographic patterning of youth vaping and youth smoking are shown in Table 1. Among all youth, 3.4% vaped, 7.4% had ever smoked, 2.3% were current smokers, and 0.9% had initiated smoking since the previous survey wave. Smoking prevalence was higher among youth who vaped, regardless of definition (ever smoked: 63.3%; current: 31.9%; initiation: 24.1%). Prevalence of youth vaping and smoking were both higher among youth whose parents vaped (vaping: 5.6%; ever smoked: 12.3%; current: 4.0%; initiation: 2.4%).

### Youth vaping and youth smoking

**Fig. 1:**
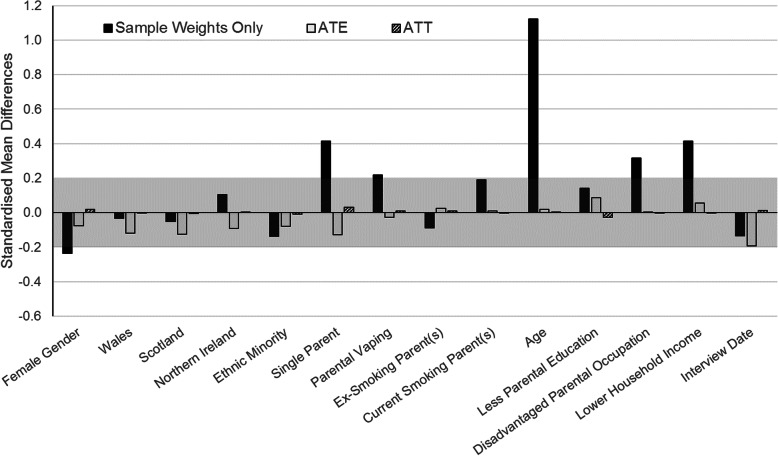
Standardised Mean Differences in Confounders Associated with Youth Vaping. The shaded band indicates the area we considered close to zero

Mean ATE weights were close to 1 (0.999) indicating stability. Figure 1 shows standardised mean differences in confounders associated with youth vaping before and after propensity weighting. Youth who vaped were more likely to be male, older, come from disadvantaged or single-parent households, and have vaping parents. Propensity weighting attenuated these differences to below the 0.2 standard deviation threshold, indicating successful balancing of confounder characteristics across exposure groups. Similar confounder balance was achieved among the sub-sample of youth used for analyses of initiation (results not shown).

Table 3 shows estimates of effects of youth vaping on youth smoking. Associations between youth vaping and ever smoking were attenuated by around two thirds when weighting for confounder differences to estimate both the ATE and the ATT. Estimates of ATEs for youth vaping on current smoking and smoking initiation were attenuated by 40 and 26% respectively relative to the sample-weighted associations. ATT estimates were attenuated by 74 and 92%. While confidence intervals for ATE and ATT estimates over-lapped, the ATT estimate for smoking initiation was considerably weaker than the ATE estimate (*p* = 0.087) and its confidence intervals over-lapped the null. The e-values for the lower confidence interval limits indicate that a set of unmeasured confounders would need to be associated with both smoking and vaping with ORs in excess of 9, independently of our measured confounders, to negate most of these effect estimates (the ATT effect estimate for initiation being the exception). Greater unmeasured confounding influences would be needed to negate the ATE estimates for current smoking and initiation than for the ATT estimates for these measures.

**Table 3:** Estimates of effects of youth vaping on youth smoking

	OR	95% CI	E-Value for OR	E-Value for lower limit
**Ever Smoked** (*n* = 3291)				
Sample weighted association	29.78	17.89–49.58	59.06	35.27
ATE	12.03	5.16–28.04	23.55	9.79
ATT	10.54	5.99–18.53	20.57	11.46
**Current Smoking (**n = 3291**)**				
Sample weighted association	37.26	19.78–70.18	74.02	39.05
ATE	22.71	8.99–57.40	44.91	17.47
ATT	10.49	5.04–21.82	20.47	9.55
**Smoking Initiation (**n = 3075**)**				
Sample weighted association	43.34	15.04–124.89	86.18	29.57
ATE	32.46	9.84–107.09	64.42	19.17
ATT	4.38	0.62–30.94	8.23	1.00

ATE estimates the average effect of vaping among all youth

ATT estimates the average effect of vaping among youth who do vape

The e-value for the OR indicates the minimum strength of association (OR) that an unmeasured confounder would need to have with both youth vaping and youth smoking to reduce this estimate to the null. The e-value for the lower limit indicates the minimum strength of association that an unmeasured confounder would need to have with both youth vaping and youth smoking for the lower limit of the confidence intervals around this estimate to cross the null (all confounders are unmeasured for the sample weighted associations)

### Parental vaping and youth smoking and vaping

**Fig. 2:**
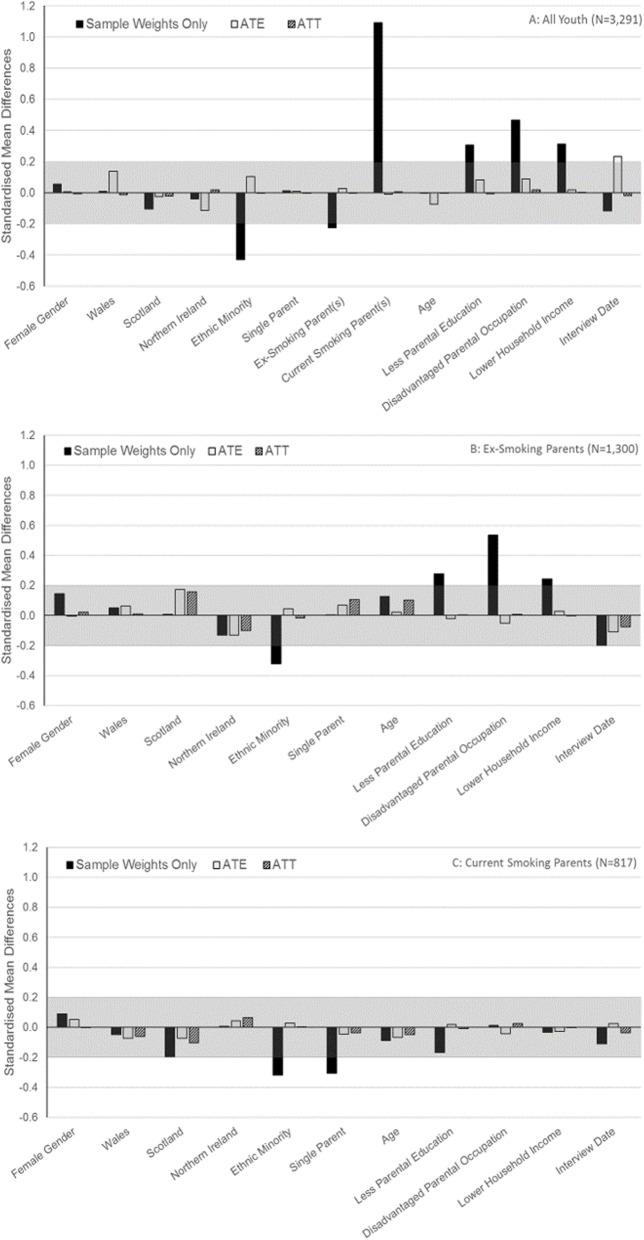
Standardised Mean Differences in Confounders Associated with Parental Vaping. The shaded bands indicate the area we considered close to zero

There were only 12 cases of youth whose parents vaped but had never smoked. This was considered insufficient information to estimate effects of parental vaping among youth whose parents never smoked, so we present results for: *all youth* combined; *youth with ex-smoking parents*; and *youth with currently smoking parents*. Mean ATE weights for parental vaping were close to 1 (1.001) indicating stability. Figure 2 shows standardised mean differences in confounders associated with parental vaping for each of these groups. Parental vaping was associated: with more parental current smoking and less parental ex-smoking, and with socioeconomic disadvantage and ethnic majority status among all youth (Fig. 2a); with ethnic majority status and socioeconomic disadvantage among youth with ex-smoking parents (Fig. 2b); and with ethnic majority status and having couple parents among youth with currently smoking parents (Fig. 2c). Propensity weights successfully balanced measured confounders, with the exception of a small residual bias in interview date among all youth such that youth whose parents vaped tended to be interviewed later. Similar balance was achieved for the sub-sample of youth used for analyses of initiation (results not shown).

Table 4 displays estimated effects of parental vaping on youth smoking and vaping. Regarding *all youth*, parental vaping only showed a clear sample weighted association with ever smoking. This was not fully explained by measured confounders in the ATE/ATT estimates. ATE and ATT estimates of the effect of parental vaping on smoking initiation were stronger than the sample weighted association, with the ATT indicating a clear effect. Nevertheless, the e-values suggested that a relatively small degree of unmeasured confounding could explain these effect estimates.

**Table 4:** Estimates of effects of parental vaping on youth smoking vaping

	All Youth				Ex-Smoking Parents				Current Smoking Parents				
	OR	95% CI	E-Value for OR	E-Value for Lower Limit	OR	95% CI	E-Value for OR	E-Value for Lower Limit	OR	95% CI	E-Value for OR	E-Value for Lower Limit	*P*-Value for difference^**a**^
**Youth Ever Smoking**				**N = 3291**				***N*** **= 1300**				***N*** **= 817**	
Sample weighted association	1.93	1.25–2.98	3.27	1.81	2.07	0.95–4.50	3.56	1.00	1.36	0.74–2.51	2.06	1.00	0.407
ATE	2.43	1.05–5.63	4.29	1.28	1.92	0.89–4.15	3.25	1.00	1.37	0.73–2.56	2.08	1.00	0.505
ATT	1.68	1.03–2.72	2.75	1.21	1.84	0.81–4.16	3.08	1.00	1.51	0.80–2.82	2.39	1.00	0.702
**Youth Current Smoking**				**N = 3291**				**N = 1300**				**N = 817**	
Sample weighted association	1.99	0.96–4.10	3.39	1.00	3.59	1.07–12.01	6.64	1.34	0.78	0.30–2.00	1.88	1.00	0.050
ATE	1.61	0.62–4.20	2.60	1.00	3.80	1.13–12.77	7.06	1.51	0.75	0.30–1.89	2.00	1.00	0.037
ATT	1.27	0.58–2.81	1.86	1.00	2.73	0.74–10.03	4.90	1.00	0.87	0.34–2.28	1.56	1.00	0.167
**Youth Smoking Initiation**				**N = 3075**				***N*** **= 1209**				***N*** **= 749**	
Sample weighted association	3.11	0.94–10.31	5.67	1.00	10.87	2.69–43.97	21.23	4.82	1.19	0.17–8.42	1.67	1.00	0.071
ATE	3.50	0.81–15.03	6.46	1.00	11.28	2.74–46.44	22.05	4.92	1.01	0.16–6.42	1.11	1.00	0.042
ATT	3.76	1.27–11.19	6.98	1.86	6.81	1.78–25.96	13.10	2.96	2.58	0.50–13.35	4.60	1.00	0.370
**Youth Vaping**				**N = 3291**				**N = 1300**				**N = 817**	
Sample weighting association	1.85	0.97–3.53	3.10	1.00	3.74	1.30–10.76	6.94	1.92	0.96	0.39–2.38	1.25	1.00	0.181
ATE	1.34	0.58–3.09	2.01	1.00	2.61	0.82–8.31	4.66	1.00	1.02	0.42–2.47	1.16	1.00	0.087
ATT	1.48	0.72–3.04	2.32	1.00	2.70	0.88–8.27	4.84	1.00	1.08	0.42–2.76	1.37	1.00	0.218

^a^P-value testing equivalence of OR for ex compared to current smoking parents

ATE estimates the average effect of parental vaping among all youth (or within strata of parental smoking)

ATT estimates the average effect of parental vaping among youth whose parents do vape (or those whose parents do vape within strata of parental smoking). The e-value for the OR indicates the minimum strength of association (OR) that an unmeasured confounder would need to have with both parental vaping and youth outcomes to reduce this estimate to the null. The e-value for the lower limit indicates the minimum strength of association that an unmeasured confounder would need to have with both parental vaping and youth outcomes for the lower limit of the confidence intervals around this estimate to cross the null (all confounders are unmeasured for the sample weighted associations)

Among *youth with ex-smoking parents* (i.e. comparing youth with ex-smoking parents who vaped versus those with ex-smoking parents who did not vape), parental vaping was associated with youth current smoking, youth smoking initiation and youth vaping. For current smoking and initiation, ATE estimates were slightly stronger than the sample weighted associations but were attenuated by 41% for youth vaping. ATT estimates were attenuated by 33–41% relative to the sample-weighted associations, but still indicated a clear effect on smoking initiation. Again, the e-values indicated that relatively little unmeasured confounding would be required to explain these effects. Among *youth with current smoking parents* none of the estimates indicated much evidence for relationships with parental vaping.

## Discussion

### Summary of findings

We found associations between vaping and smoking among youth, and some associations between parental vaping and youth smoking and youth vaping. Effects of youth vaping on youth smoking were estimated using propensity weights to balance measured confounders between youth who did and did not vape. Common liabilities related to these measured confounders seemed to explain considerable proportions of the association between youth vaping and smoking. Nevertheless, unmeasured confounders (e.g. beliefs, values, personality, or sibling/peer smoking) would need to have quite strong independent associations (ORs generally of magnitude 9 or more) with both smoking and vaping to explain the residual relationship. Unmeasured confounding of this magnitude is possible, though less likely, in the form of a single strong confounding factor, but could be feasible as an aggregate effect from a set of weaker confounders [34]. Particularly novel in our findings is the suggestion of heterogeneity in the relationship between smoking and vaping in youth. In comparison to estimates of average effects among all youth, estimates of effect among youth who vaped were weaker and more easily explained by unmeasured confounding, especially for initiation of smoking.

In relation to effects of parental vaping on youth smoking and youth vaping, while some estimates still indicated effects of parental vaping after adjusting for measured confounders, a relatively small degree of unmeasured confounding (e.g. if ex-smoking parents who vape have different smoking histories from those who do not) would suffice to explain these estimates. We found no support for our postulation that the effects of parental vaping would be weaker or reversed when parents had completely switched from cigarettes to e-cigarettes, compared to parents using both. Contrarily, parental vaping was most strongly associated with youth smoking and vaping among youth whose parents were ex-smokers, with little evidence of associations with parental vaping among those whose parents currently smoked (effects were not estimated among parents who had never smoked as there were too few who vaped).

### Limitations

Our effect estimates assume a causal direction going from youth vaping to youth smoking (chosen as the most concerning direction of effect), but it is important to emphasise that these estimates could be either partially or completely accounted for by effects of youth smoking on youth vaping (i.e. reverse causation, e.g. youth using e-cigarettes as a smoking cessation aid) [8], especially as others have shown longitudinal effects in both directions [22]. This would include our measure of smoking initiation, because initiation within the past year could have led to vaping. However, reverse causation is less likely for the initiation estimates than for those relating to current smoking, as youth who have been smoking for longer would be excluded. Reverse causation could also explain the residual effects estimated for parental vaping on youth smoking and vaping (e.g. if youth behaviour prompts parents to take up vaping).

Furthermore, while the present tense “Do you ever” wording of the question on vaping in this survey should primarily identify current vaping, the wording is ambiguous and may feasibly have been interpreted by some respondents as “Have you ever used electronic cigarettes?” Our measures of vaping could therefore include both very infrequent and/or ever use in addition to current vaping. This question also does not distinguish between different types of e-cigarette/vaping devices, or motivations for vaping [35, 36], and our estimates may have changed if these factors could have been taken into account. Additionally, the data focus on youth in the age range of 10–15 years. The prevalence of both smoking and vaping in this age group was very low, contributing to large magnitude odds ratios and wide confidence intervals, and the associations and effects could differ among older youth, as they increasingly adopt more adult behaviours.

### Meaning & Implications

Our findings align with others showing strong associations between youth smoking and vaping [2, 6, 8, 11–17, 21, 22, 37]. We were able to explain much of this association with a relatively limited set of measured confounders and, as noted above, the residual effect estimates could be at least partially explained by unmeasured confounding and/or reverse causation. Taken together with other evidence such as observed increases in youth smoking after implementation of e-cigarette sale restrictions [38–40] and continued declines in youth pro-smoking attitudes while youth vaping has been rising [41], it seems that even if there is an effect whereby vaping increases risk for smoking, it is unlikely to be the primary or dominant explanation for associations between the two, and ongoing concerns about such effects should be abated.

More importantly, the weaker estimates we found for ATT compared to ATE effects suggest that any effect of youth vaping on youth smoking may be weaker for youth already pre-disposed to vaping by background factors. This is consistent with another study that found stronger effects of vaping among youth with no intention to smoke [23]. Thus, effects of vaping on smoking could become more salient and important if vaping were adopted much more widely and by a broader range of youth, as opposed to the current low prevalence in the UK. Governments may want to prioritise preventing wider adoption, e.g. with e-cigarette age-of-sale and advertising restrictions (actions that many public health actors agree on [42]), over changing or stopping current youth vaping behaviour (e.g. via an all-out ban).

Perhaps propensities for vaping and smoking are similar enough that vaping has little additional impact where propensity for smoking is already high. Indeed, one theoretical explanation for why young people may transition from vaping to smoking is that vaping provides experience and training in the social performance of a similar behaviour, which might otherwise be unfamiliar [9]. Such first-hand experience could come from infrequent or even singular experiences with vaping and could plausibly be more important for young people without a background predisposition to use, who may have less experience of seeing others smoke or vape. Mechanisms for why young people would begin vaping before smoking include e-cigarettes being viewed as less harmful, more acceptable, having attractive flavours, and being easier to conceal [9]; these mechanisms could also be more salient for those without a background propensity for use. Similar mechanisms might explain why parental vaping had little impact among youth whose parents currently smoked; risk for use might already be high enough in this group that any weak effect of parental vaping has little further influence.

## Conclusions

Mounting evidence supports the view that e-cigarettes are substantially less harmful than traditional cigarettes [1, 16, 43–46], and that they can aid successful smoking cessation [3, 16, 35, 47–49]. While potential public health benefits of e-cigarettes should be weighed against possible detriments [25, 50], we found little evidence to support concerns about negative impacts on youth smoking or vaping behaviour. While we could not rule out effects whereby youth vaping increases risk for smoking, associations were substantially attenuated by measured confounders, indicating support for common liabilities, and the residual associations observed could potentially be explained by reverse causation and/or strong unmeasured confounding. Estimates of effects of youth vaping on youth smoking were stronger among youth not already predisposed to vaping, so it may be important to prevent wider-scale adoption of vaping by youth. There was some evidence for effects of parental vaping on youth smoking and vaping, particularly among youth whose parents were ex- rather than current smokers, but relatively weak unmeasured confounding would suffice to explain these effects. Further monitoring and research may be advisable, but evidence here did not strongly support potential concerns regarding negative impacts on child behaviour if smoking parents use e-cigarettes as cessation aids.

## Data Availability

The datasets analysed during the current study are available from the UK Data Service repository. DOI: 10.5255/UKDA-SN-6614-13

## Abbreviations

ATE: Average treatment effect
ATT: Average treatment effect among the treated
CI: Confidence interval
E-Cigarettes: Electronic cigarettes
GCSE: General certificate of secondary education
NS-SEC: National statistics socioeconomic classification
OR: Odds ratio
SEP: Socioeconomic position
UK: United Kingdom

## References

[1] McNeill A, Brose LS, Calder R, Hitchman SC, Hajek P, McRobbie H (2015). E-cigarettes: an evidence update.

[2] Chapman CS, Wu L (2014). E-cigarette prevalence and correlates of use among adolescents versus adults: a review and comparison. J Psychiatr Res.

[3] McNeill A, Brose LS, Calder R, Bauld L, Robson D (2018). Evidence review of e-cigarettes and heated tobacco products 2018.

[4] McNeill A, Brose LS, Calder R, Bauld L, Robson D (2019). Vaping in England, an evidence update, February 2019. A report commissioned by Public Health England.

[5] Grana RA (2013). Electronic cigarettes: a new nicotine gateway?. J Adolesc Health.

[6] Leventhal AM, Strong DR, Kirkpatrick MG, Unger JB, Sussman S, Riggs NR, Stone MD, Khoddam R, Samet JM, Audrain-McGovern J (2015). Association of electronic cigarette use with initiation of combustible tobacco product smoking in early adolescence. JAMA.

[7] Bell K, Keane H (2014). All gates lead to smoking: the ‘gateway theory’, e-cigarettes and the remaking of nicotine. Soc Sci Med.

[8] Etter J-F (2018). Gateway effects and electronic cigarettes. Addiction.

[9] Schneider S, Diehl K (2016). Vaping as a catalyst for smoking? An initial model on the initiation of electronic cigarette use and the transition to tobacco smoking among adolescents. Nicotine Tob Res.

[10] Kandel ER, Kandel DB (2014). A molecular basis for nicotine as a gateway drug. N Engl J Med.

[11] Dutra LM, Glantz SA (2014). Electronic cigarettes and conventional cigarette use among US adolescents: a cross-sectional study. JAMA Pediatr.

[12] Hughes K, Bellis MA, Hardcastle KA, McHale P, Bennet A, Ireland R, Pike K (2015). Associations between e-cigarette access and smoking and drinking behaviours in teenagers. BMC Public Health.

[13] Scottish Government (2017). Scottish Schools Adolescent Lifestyle and Substance Use Survey (SALSUS) 2015: Six key facts about e-cigarette use.

[14] Krishnan-Sarin S, Morean ME, Camenga DR, Cavallo DA, Kong G (2015). E-cigarette use among high school and middle school adolescents in Connecticut. Nicotine Tob Res.

[15] Treur JL, Rozema AD, Mathijssen JJ, van Oers H, Vink JM (2018). E-cigarette and waterpipe use in two adolescent cohorts: cross-sectional and longitudinal associations with conventional cigarette smoking. Eur J Epidemiol.

[16] National Academies of Sciences Engineering & Medicine (2018). Public Health Consequences of E-Cigarettes.

[17] Green MJ, Hilton S (2018). Applying recommended evidence standards to understand the impact of e-cigarettes on youth smoking and reporting of weak scientific evidence. Addiction.

[18] Leventhal AM, Stone MD, Andrabi N (2016). Association of e-cigarette vaping and progression to heavier patterns of cigarette smoking. JAMA.

[19] Conner M, Grogan S, Simms-Ellis R, Flett K, Sykes-Muskett B, Cowap L, Lawton R, Armitage CJ, Meads D, Torgerson C (2018). Do electronic cigarettes increase cigarette smoking in UK adolescents? Evidence from a 12-month prospective study. Tob Control.

[20] Best C, Haseen F, Currie D, Ozakinci G, MacKintosh AM, Stead M, Eadie D, MacGregor A, Pearce J, Amos A (2018). Relationship between trying an electronic cigarette and subsequent cigarette experimentation in Scottish adolescents: a cohort study. Tob Control.

[21] Soneji S, Barrington-Trimis JL, Wills TA, Leventhal AM, Unger JB, Gibson LA, Yang J, Primack BA, Andrews JA, Miech RA (2017). Association between initial use of e-cigarettes and subsequent cigarette smoking among adolescents and young adults: a systematic review and meta-analysis. JAMA Pediatr.

[22] East K, Hitchman SC, Bakolis I, Williams S, Cheeseman H, Arnott D, McNeill A (2018). The association between smoking and electronic cigarette use in a cohort of young people. J Adolesc Health.

[23] Barrington-Trimis JL, Urman R, Berhane K, Unger JB, Cruz TB, Pentz MA, Samet JM, Leventhal AM, McConnell R. E-Cigarettes and Future Cigarette Use. Pediatrics. 2016;138(1):e20160379. 10.1542/peds.2016-0379 PMC4925085 27296866

[24] Tyas SL, Pederson LL (1998). Psychosocial factors related to adolescent smoking: a critical review of the literature. Tob Control.

[25] Hilton S, Weishaar H, Sweeting H, Trevisan F, Katikireddi SV (2016). E-cigarettes, a safer alternative for teenagers? A UK focus group study of teenagers' views. BMJ Open.

[26] Lozano P, Arillo-Santillán E, Barrientos-Gutíerrez I, Reynales Shigematsu LM, Thrasher JF (2019). E-cigarette social norms and risk perceptions among susceptible adolescents in a country that bans E-cigarettes. Health Educ Behav.

[27] Austin PC (2011). An introduction to propensity score methods for reducing the effects of confounding in observational studies. Multivar Behav Res.

[28] University of Essex, Institute for Social and Economic Research, NatCen Social Research, Kantar Public. Understanding Society: Waves 1–8, 2009–2017 and Harmonised BHPS: Waves 1–18, 1991–2009. 11th Edition edn. Colchester: UK Data Service; 2018.

[29] Knies G (2017). Understanding society: waves 1–7, 2009–2016 and harmonised BHPS: waves 1–18, 1991–2009, user guide.

[30] Seaman SR, White IR, Copas AJ, Li L (2012). Combining multiple imputation and inverse-probability weighting. Biometrics.

[31] Austin PC, Stuart EA (2015). Moving towards best practice when using inverse probability of treatment weighting (IPTW) using the propensity score to estimate causal treatment effects in observational studies. Stat Med.

[32] Cole SR, Hernán MA (2008). Constructing inverse probability weights for marginal structural models. Am J Epidemiol.

[33] Clogg CC, Petkova E, Haritou A (1995). Statistical methods for comparing regression coefficients between models. Am J Sociol.

[34] VanderWeele TJ, Ding P (2017). Sensitivity analysis in observational research: introducing the E-value. Ann Intern Med.

[35] Villanti AC, Feirman SP, Niaura RS, Pearson JL, Glasser AM, Collins LK, Abrams DB (2018). How do we determine the impact of e-cigarettes on cigarette smoking cessation or reduction? Review and recommendations for answering the research question with scientific rigor. Addiction.

[36] Hitchman SC, Brose LS, Brown J, Robson D, McNeill A (2015). Associations between E-cigarette type, frequency of use, and quitting smoking: findings from a longitudinal online panel survey in Great Britain. Nicotine Tob Res.

[37] Park J-Y, Seo D-C, Lin H-C (2016). E-cigarette use and intention to initiate or quit smoking among US youths. Am J Public Health.

[38] Friedman AS (2015). How does electronic cigarette access affect adolescent smoking?. J Health Econ.

[39] Pesko MF, Currie JM (2016). The effect of E-cigarette minimum legal Sale age Laws on traditional cigarette use and birth outcomes among pregnant teenagers. National Bureau of Economic Research Working Paper Series.

[40] Dhaval D, Bo F, Pesko MF (2017). The Effects of E-Cigarette Minimum Legal Sale Age Laws on Youth Substance Use. National Bureau of Economic Research Working Paper Series.

[41] Hallingberg B, Maynard O, Bauld L, Brown R, Gray L, Lowthian E, MacKintosh A, Moore L, Munafò M, Moore G (2020). Have e-cigarettes renormalised or displaced youth smoking? Results of a segmented regression analysis of repeated cross sectional survey data in England, Scotland and Wales. Tobacco Control.

[42] Weishaar H, Ikegwuonu T, Smith KE, Buckton CH, Hilton S (2019). E-cigarettes: a disruptive technology? An analysis of health actors’ positions on E-cigarette regulation in Scotland. Int J Environ Res Public Health.

[43] Abrams DB, Glasser AM, Pearson JL, Villanti AC, Collins LK, Niaura RS (2018). Harm minimization and tobacco control: reframing societal views of nicotine use to rapidly save lives. Annu Rev Public Health.

[44] Hajek P, Etter J-F, Benowitz N, Eissenberg T, McRobbie H (2014). Electronic cigarettes: review of use, content, safety, effects on smokers and potential for harm and benefit. Addiction.

[45] Shahab L, Goniewicz ML, Blount BC (2017). Nicotine, carcinogen, and toxin exposure in long-term e-cigarette and nicotine replacement therapy users: a cross-sectional study. Ann Intern Med.

[46] Stephens WE (2018). Comparing the cancer potencies of emissions from vapourised nicotine products including e-cigarettes with those of tobacco smoke. Tob Control.

[47] Beard E, West R, Michie S, Brown J. Association between electronic cigarette use and changes in quit attempts, success of quit attempts, use of smoking cessation pharmacotherapy, and use of stop smoking services in England: time series analysis of population trends. BMJ. 2016;354:i4645. 10.1136/bmj.i4645 27624188

[48] Benmarhnia T, Pierce JP, Leas E, White MM, Strong DR, Noble ML, Trinidad DR (2018). Can E-cigarettes and pharmaceutical aids increase smoking cessation and reduce cigarette consumption? Findings from a nationally representative cohort of American smokers. Am J Epidemiol.

[49] Li J, Hajek P, Pesola F, Wu Q, Phillips-Waller A, Przulj D, Myers Smith K, Bisal N, Sasieni P, Dawkins L (2020). Cost-effectiveness of e-cigarettes compared with nicotine replacement therapy in stop smoking services in England (TEC study): a randomized controlled trial. Addiction.

[50] Eissenberg T, Bhatnagar A, Chapman S, Jordt S-E, Shihadeh A, Soule EK (2020). Invalidity of an oft-cited estimate of the relative harms of electronic cigarettes. Am J Public Health.

